# The Clinical Outcome of Perforator Based Sural Artery and Propeller Flaps in Reconstruction of Soft Tissue of Extremities

**DOI:** 10.29252/wjps.8.1.3.

**Published:** 2019-01

**Authors:** Subha Dhua, Sankhe Manashree, Bal Gangadhar Tilak

**Affiliations:** Department of Plastic and Reconstructive Surgery, Vydehi Institute of Medical Science and Research Centre, Bangalore, India

**Keywords:** Propeller flap, Subcutaneous pedicled propeller flap, Perforator pedicled propeller flap

## Abstract

**BACKGROUND:**

The reconstructive options for the soft tissues in extremities present serious challenges due to thin non-expendable soft tissues and predisposition to massive edema formation, thus frequently requiring flap cover. This study was undertaken to assess the outcome of a modified version of the sural artery flap with that of propeller perforator flaps for the reconstruction of lower extremities, particularly the heel defects.

**METHODS:**

This prospective study was conducted on 40 consecutive patients, of which 20 treated with sural artery flap and another 20 with perforator based propeller flap cover for soft tissue reconstruction in extremities based on predefined inclusion criteria. The clinical outcome of the flap was assessed after three months.

**RESULTS:**

Inclusion of the posterior tibial perforators (along with the sural artery and the peroneal artery) was shown to enhance the flap territory. The raising of the flap was quick with minimal blood loss and the modified flap had a wide arc of rotation for reconstruction of the heel defects. The younger patients regained sensation on the flap earlier, while the durability of the fasciocutaneous flap was excellent in the heel weight bearing areas. The success rate was 95% flap take.

**CONCLUSION:**

Careful selection of the perforator and optimal designing of the flap result in favorable outcomes with the use of local perforator flaps for reconstruction in extremities. It provides predictable clinical outcomes with minimal donor site morbidity, is cost-effective, relatively easy technique and requires no special microsurgical setup or instruments.

## INTRODUCTION

The first description of reconstructive surgery can be traced back in India, perhaps two millennia, while in Europe to the fifteenth century. Reference to the reconstruction of missing parts of the face, and to the treatment of mandibular fractures, they are found in Egyptian papyri and in the Hindu Susruta.^1 ^In the sixteenth century, the first text book entitled “De Curtorum Chirurgia per Insitionem” was published by Tagliocozzi, Professor of Surgery in Bologna in 1597.^1^ In 1854,^1^ the first attempt was undertaken to bring together all the methods, and advances made in the field of reconstructive surgery in a publication entitled ‘Practical Essays in Plastic Surgery’. During the first World War (1914-1918), Harold Gillies treated dreadful facial, and other injuries caused by sophisticated weapons of that time, and published the results of his work in his classic book entitled ‘The Plastic Surgery of the Face’, who described the tube pedicle flap and commented that heel was difficult to heal.^2^

 Several researchers described the use of free flap.^3^^-^^5^ In 1981, the development of fasciocutaneous flaps was introduced and in 1990, perforator based flaps came to be in use.^6^ In 1983,^7^ and 1994^8^ in anatomic dissection and radiographic studies, the blood supply to the sural artery flap was demonstrated. In 1993, Wu *et al.* studied twenty legs of ten cadavers to establish the vascular anatomy of the posterior tibial vessels including the number, size and distribution of the direct cutaneous and direct muscle branches of the posterior tibial vessels.^9^ Flaps of the posterior side of the lower leg were reported by Masquelet *et al.* (1992),^10^ Hasegawa *et al.* (1994),^11^ Hyakusoku* et al.* (1994),^12^ Oberlin *et al.* (1995),^13^ and Rajacic *et al.* (1996)^14^ corresponded to the distally based lesser saphenous-sural V-NAF flap. They observed that most of the venous blood returned first to the trunk of the lesser saphenous vein.^10^^-^^14^

The observations of Imanishi *et al.* (1999) showed that direct reflux through the valves in the lesser saphenous vein did not occur.^15^ Small concomitant veins were found by them along both sides of the lesser saphenous vein and considered to be venae concomitants of accompanying arteries of the vain. Skin grafted muscle flaps for defects of lower third of the leg were popularized in 1966. Later Ponten (1981)^6^ showed the advantages of fasciocutaneous flaps and was later confirmed by Barclay *et al.* (1982).^16^ In 1995, the anastomotic network between sural artery and peroneal septocutaneous perforators in cadaveric dissections was demonstrated.^17^ In 1997, Zang, *et al.* described postero-lateral and postero-medial distally based island fasciocutaneous flaps for lower one-third of leg defects.^18^

In 2001, Lefourn *et al.*,^19^ Al. Qattan^20^ and Mueller *et al.*^21^ described a fasciomuscular sural artery flap in which a sleeve of gastocnemius muscle was included at the sural artery which passed deep between the two heads of gastrocnemius flap for better vascularity. Later, the cross-leg flaps and then the local fasciocutaneous flaps came into use. The coverage of the heel defects with sural artery flap underwent some modifications over a period of time which revolutionized the management of heel defects. The present studies have modified the flap with the inclusion of the posterior perforators and its fasciocutaneous territory and including a sleeve of gastrocnemius muscle only where, the median sural artery is crossing between the two heads of the gastrocnemius muscle. 

The quest of plastic surgeons for the ideal flap coverage to be used in soft tissue defect reconstruction has led to a steady evolution of reconstructive surgery. Soft tissue reconstruction is used to define the various surgical techniques devised to cover raw area from a post traumatic event, defect formed from excision of scar/oncological disease, post-burn contracture excision defects or from incision wounds. Soft tissue reconstruction can be a simple process or it can be an extensive and complicated surgical procedure, depending on the location and the type of reconstructive technique being used. This study was undertaken to assess the outcome of a modified version of the sural artery flap with that of propeller perforator flaps for the reconstruction of lower extremities, particularly the heel defects.

## MATERIALS AND METHODS

The institutional ethical committee clearance was obtained for the study, which is attached as Annexure 1. Those patients who fitted the study inclusion criteria were required to provide informed consent in the prescribed format of the institution which was attached as Annexure 2. The study was a prospective study conducted on 40 consecutive patients, of which 20 were treated with sural artery flap and another 20 with perforator based propeller flap cover for soft tissue reconstruction in extremities based on predefined inclusion criteria. Demographic and clinical details about the soft tissue defect, details of the perforator propeller flap cover used and post-operative events in details were noted and clinical outcome of the flap was assessed on the basis of 3 parameters including (i) percentage of flap surface area survival, (ii) healing of the skin margins of the flap after suture removal and (iii) presence or absence of complications. Patients were followed up for a period of three months from the time of discharge.

Inclusion criteria were considered as 1) patients to be between the ages of 12–70 years, with soft tissue defects over extremities requiring reconstruction due to acute or chronic trauma, 2) post-tumor excision, 3) post-burn contracture reconstruction, and 4) post infective raw area. Exclusion criteria were regarded as patients with 1) anemia, 2) acute infection, 3) peripheral vascular disease, 4) uncontrolled diabetes mellitus, and liver disease, 5) undergoing radiation, 6) pregnant women, and 7) heavy smokers (more than 25 cigarettes/day). The institutional ethical committee clearance was obtained for the study. Those patients who fitted the study inclusion criteria and gave an informed written consent for the study were enrolled. The predesigned proforma was maintained for all patients. Preoperative Doppler assessment was done for each patient and findings noted in proforma. 

The patients were then prepared for surgery. Pre-anesthesia check-up was done for each patient and written informed consent was taken for surgery. Basic lab investigations like hemoglobin (Hb), fasting blood sugar (FBS) or random blood sugar (RBS), blood urea, serum creatinine and electrocardiogram (ECG) were conducted routinely for all patients. Chest X-ray was undertaken when indicated. Xylocaine test dose was done for all patients pre-operatively. All patients were pre-medicated with a premedication, ranitidine (150 mg) and diazepam (5 mg) tablets orally the night before surgery. 

Anesthesia machine, circuits, emergency drugs and equipment and monitors were checked before starting the case. The monitors used were electrocardiogram (ECG), pulse oxymeter, and noninvasive blood pressure (NIBP). Invasive vascular access was secured depending on the need. IV line secured using 18-gauge cannula and ringer lactate infusion started. Base line blood pressure, heart rate and respiratory rate were noted. On arrival in the operating room, patients were preloaded with lactated ringer’s solution (15 ml/kg). Patient was given regional or general anesthesia as indicated. The 42 surgeries were started after painting and draping were performed using strict aseptic precautions. 

**Fig. 1:**
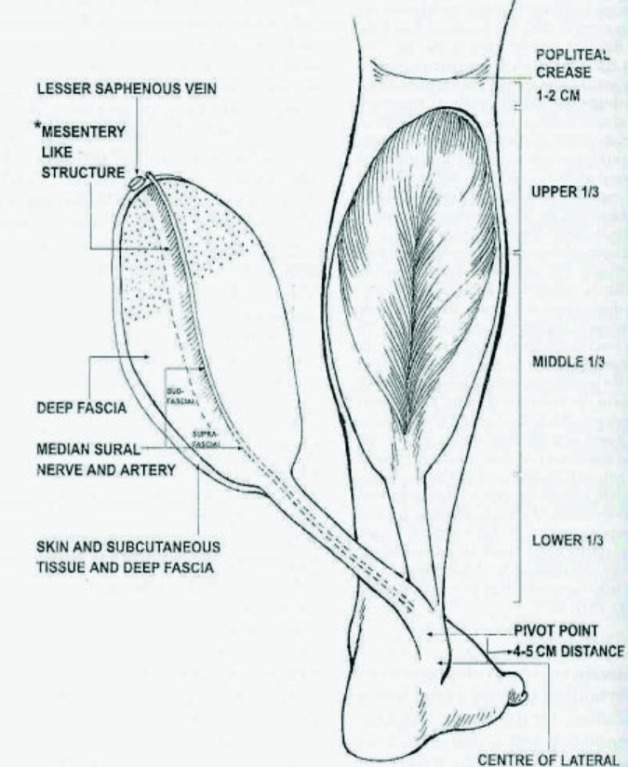
Diagrammatic presentation of flap with anatomy

The superficial sural artery was marked out with the aid of a Doppler probe in the midline of calf one palm breadth below the knee joint crease. A pattern of the defect was cut out and the flap was planned in reverse dimensions and then marked out with the help of the pattern. An island subcutaneously pedicled fasciocutaneous flap based on the median superficial sural artery and the distal perforators of the peroneal and posterior tibial artery was harvested. Authors in this studies included as many superficial veins as possible in the subcutaneous pedicle. A diagrammatic presentation of the flap pointing surgical anatomy in placed at Figure 1; the shaded area depicts the proximal extension of flap territory in upper third of leg, the mesentery like structure connects sural neuro-vascular structures to deep fascia.

**Fig. 2:**
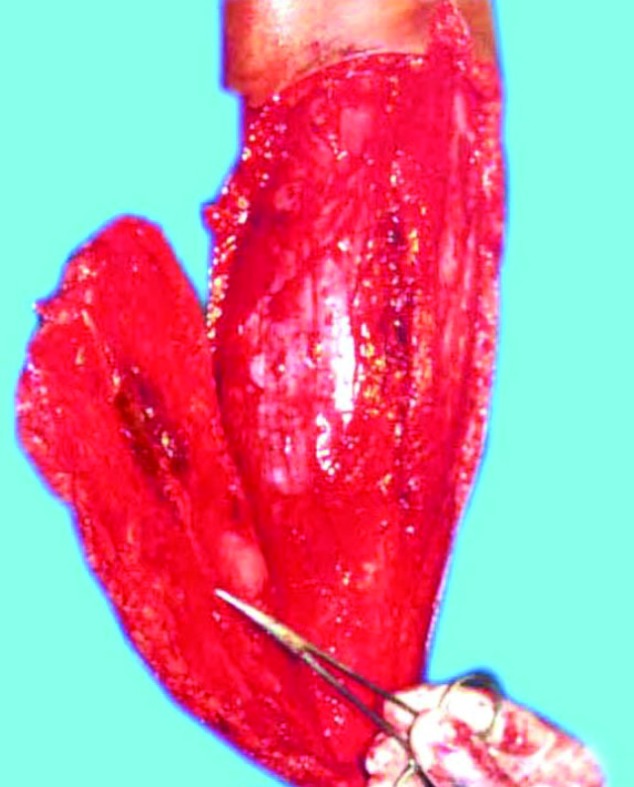
Harvesting of sural artery flap

**Fig. 3:**
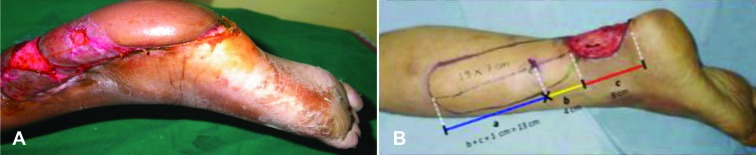
A) Flap settling in perforator propeller flap marking. B) The markings and calculations of the proximal limit of flap

The incision was first made on the proximal border of the flap, where the sural artery, sural nerve and the short saphenous vein were identified, divided and ligated. Incision was then made all around and the flap elevated subfacially (Figure 2). It has been found that sometimes the sural artery runs between two heads of gastrocnemius muscle and only in such situations, a sleeve of gastrocnemius muscle was taken along with the flap to safeguard the sural artery. The skin paddle remained as per surgical defect, whereas the fascial paddle was in concurrence with the vascular pedicle. The sural artery was then cannulated and the flap was thus harvested and then inset into the defect (Figure 3A and B). The island part of the flap donor area was resurfaced with skin graft and the donor area distal to this was closed primarily with the adjacent skin flaps. The limb was immobilized in a Plaster-Of-Paris cast leaving the flap and the vascular pedicle free from pressure.

At first, the tissue defect was prepared. Dimensions of the soft tissue defect were recorded in centimeters along the horizontal and vertical dimensions. Using a handheld Doppler ultrasound scanner (8 MHz probe), the location of the strongest perforator along the selected artery was reconfirmed with the preoperative Doppler marking. Appropriate provisional flap design was planned based on the dimensions of the soft tissue defect and the perforator location using the following method. The axial length of the defect was measured in cm and defined as value C. Next, the distance between the perforator and the distal edge of the defect was measured in cm and defined as value B. The value of (C+B) added by 1 cm was then defined as A. This value A (in cm) formed the proximal limit of the flap. 

For perforator propeller flap marking, at first the tissue defect was prepared. Dimensions of the soft tissue defect were recorded in centimeters along the horizontal and vertical dimensions. Using a handheld Doppler ultrasound scanner (8 MHz probe), the location of the strongest perforator along the selected artery was reconfirmed with the pre-operative Doppler marking. Appropriate provisional flap design was planned based on the dimensions of the soft tissue defect and the perforator location using the following method. At first, axial length of the defect was measured in cm and defined as value C. 

Next, the distance between the perforator and the distal edge of the defect was measured in cm and defined as value B. The value of (C+B) added by 1 cm was then defined as A. This value A (in cms) formed the proximal limit of the flap. C â€“ Axial length of defect (in centimeters), B â€“ Distance of perforator to distal edge of defect (in centimeters), A=(C+B)+1 centimeter. Width of the flap was determined by the width of defect added by 1 cm (0.5 cm on either lateral side), with the lateral distance equally divided on either side of the perforator. This determined the proximal flap width.

**Fig. 4:**
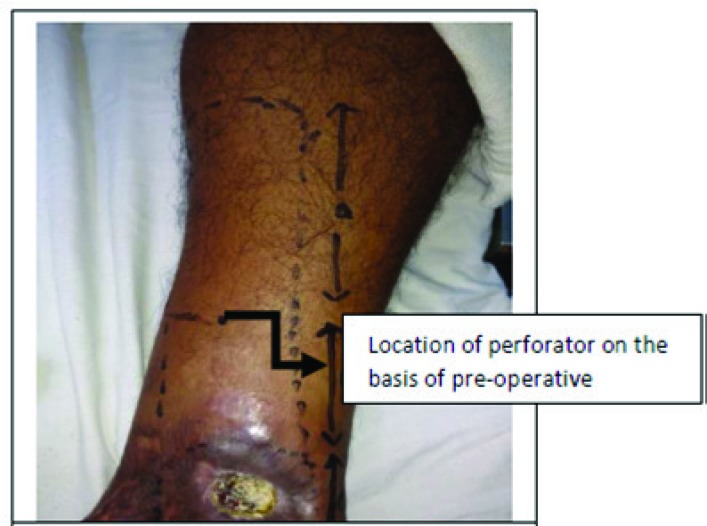
Post-burn ulcer over medial aspect of left angle

**Fig. 5:**
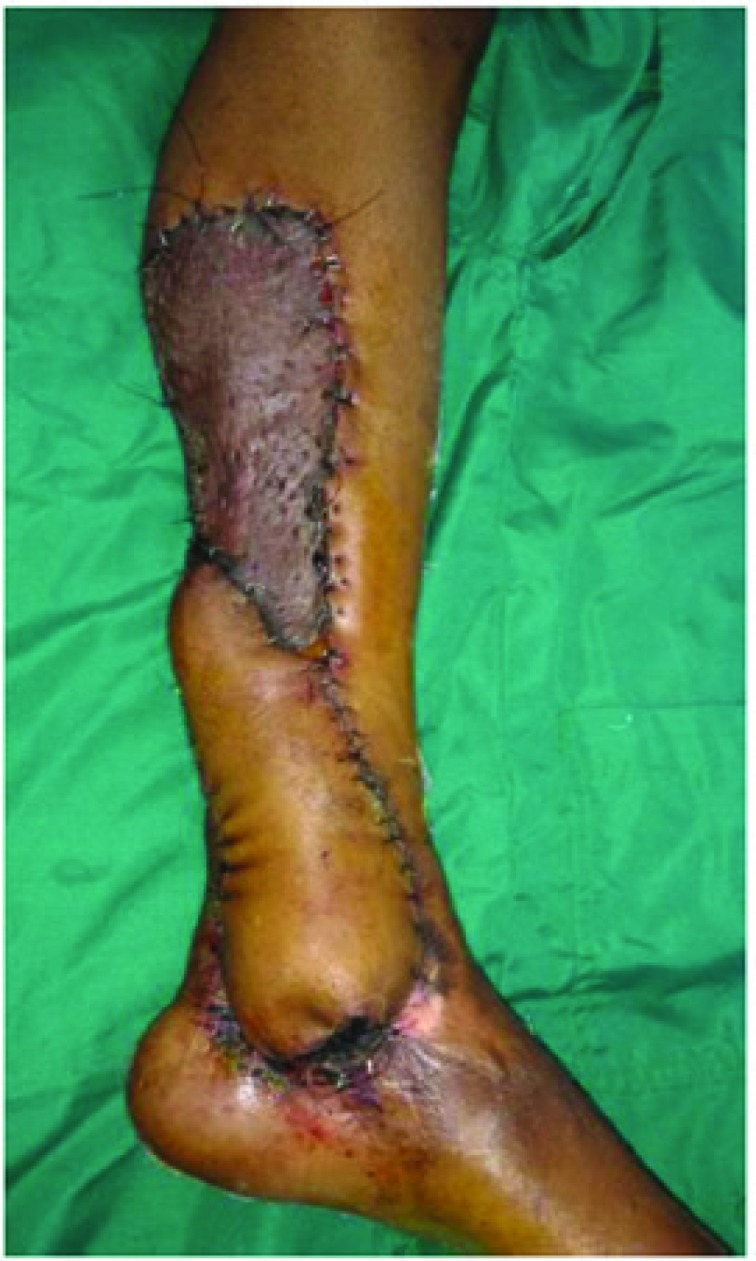
Flap islanded and transposed

**Fig. 6:**
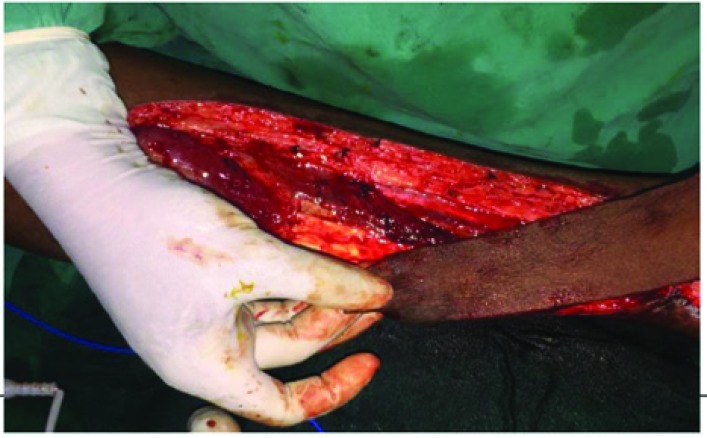
Rotation of flap by 180^o^ to cover defect in distal third of the leg

Formula for calculating Proximal Limit of Flap (Figures 4-6). C–Axial length of defect (in centimeters), B–Distance of perforator to distal edge of defect (in centimeters), A=(C+B)+1 centimeter. Width of the flap was determined by the width of defect added by 1 cm (0.5 cm on either lateral side), with the lateral distance equally divided on either side of the perforator. This determined the proximal flap width. Planning in reverse was utilized to design a diamond shaped flap over the flexion contracture according to the size of the defect that was created following the release of contracture and the normal skin available at the center of contracture. The flap could be designed into a bi-lobed, tri-lobed or multi-lobed design.

The defect area was measured and marked over the adjacent normal skin flap elevated based on at least 1 cm of subcutaneous tissue. Dissection was done gradually by teasing the subcutaneous tissue, taking care not to damage the underlying perforators, up to a level where the flap was comfortably rotated into the defect. The flap thus raised was rotated by 90°, so that the longitudinal axis was now in the transverse axis and vice versa. This rotation took place only at the peripheries of the flap. The flap once rotated was able to cover the defect. Data were analyzed and limited descriptive statistical analyses were performed using the SPSS statistical package (version 21 for Windows, Chicago, IL, USA). Demographic data were matched using the percentages and mean. 

## RESULTS 

**Fig. 7:**
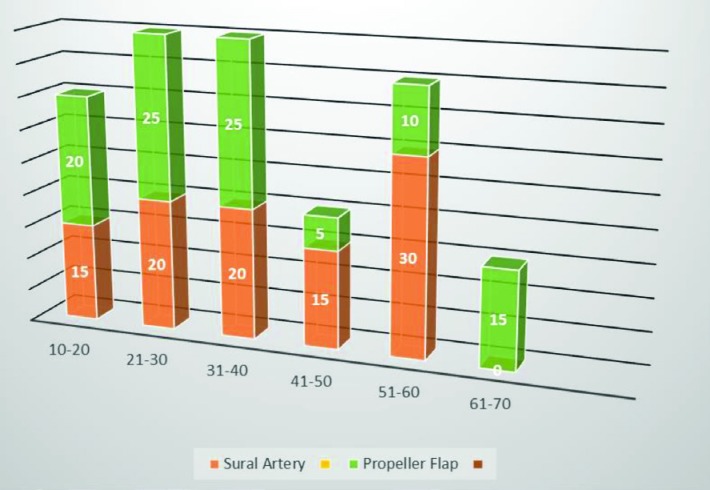
Gender distribution

Most of the patients had favorable post-operative recovery without development of any complications. The minor complications were managed with antibiotics and conservative methods. Regarding clinical profile, 20 patients with heel defect and 20 patients with soft tissue defects over lower extremities requiring reconstruction with propeller flap cover were included in the study. Results using collected clinical data were presented in percentile form. The age range of the patients included was between 1–70 years (Figure 7). Most of the patients were in the age group of 21-40 years. 

**Fig. 8:**
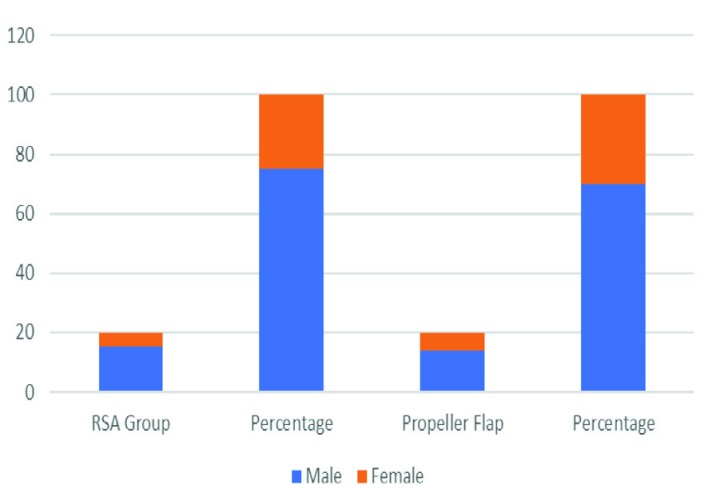
Distribution of cases according to etiology

**Fig. 9:**
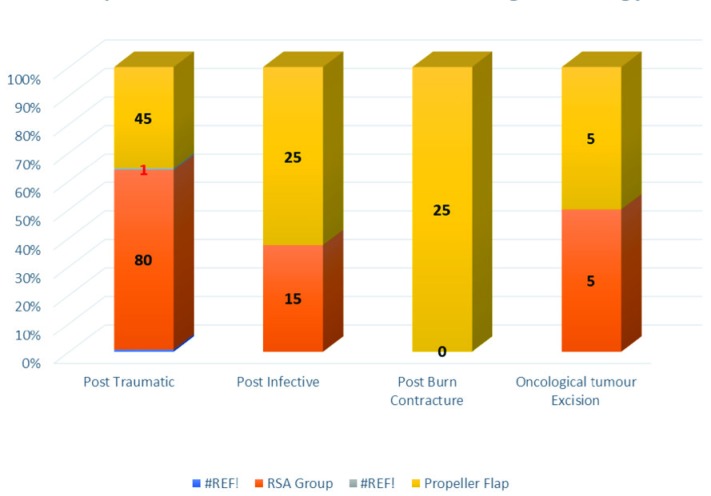
Distribution of cases according to site of tissue defect

Under the RSA group, there were 15 male patients and 5 female patients, while in the propeller flap group there were 14 male and 6 female patients. Figure 8 shows the gender distribution of the patients of the two groups. In the present studies, the most common etiology for both the groups were post-traumatic followed by post-infective groups (Figure 9). The most common site of soft tissue defect in both groups was that of the lower limb and in the RSA group that were all restricted to lower limb only. In the RSA group, the size of tissue defects ranged from 6x6 cm to 8x15 cm and in the propeller flap group ranged from 4×3 cm to 9×5.5 cm.

In the RSA group, split skin grafting (SSG) was required in all the 20 cases, while in the propeller flap group, the requirement of SSG was in 15 cases and the rest 5 cases had primary closer of the donor sites. In the RSA group, there was only minor complications with marginal necrosis and was controlled by antibiotics and settled with time. In the propeller flap group, there were also minor complications in three patients and were managed conservatively. On the basis of the complication studies, the clinical outcome of the flap survival was classified as: a) complete flap success, b) flap success with complications, and c) flap failure. Out of the 20 patients in the RSA group, 12 patients had complete flap success, while 7 cases had flap success with complications and there was 1 flap failure and in the propeller flap group, 17 patients had complete success, cases had flap success with complication and there was no failure in this group. 

## DISCUSSION

In the RSA groups, the early designs of fasciocutaneous flaps extending along the vertical axis of the leg with a proximal pedicle^6^ have been modified by the use of transverse flaps with a lateral or medial pedicle,^22^ and lastly with a distal pedicle.^23^ Present studies also took note of the fact that the blood supply to the fasciocutaneous flap was provided by the perforators from underlying muscle, septocutaneous perforators, and axial vessels.^24^ Perforators system in the lower extremity are the clinical bases of designing fasciocutaneous flap.^25^

The distally based superficial sural artery island flap was vascularized by a median superficial sural artery with reverse flow as this artery takes septocutaneous perforators from peroneal and tibial arteries in the distal part of the leg. Additionally, the sural nerve has an intrinsic arterial system. This system anastomoses freely in the superficial plexus; combinations of these systems are used to perfuse the distally based superficial sural artery island flap. Present studies used this knowledge of blood supply to the flap while designing our reliable and useful flap. We were, therefore, successful in ensuring 95% flap take. Inclusion of posterior tibial artery perforator along with sural artery and peroneal artery perforators has enabled us to cover wide defects.^26^^-^^28^

In this study, twenty patients with soft tissue defects present over extremities needing flap cover were included. Most patients were between the age group from 21 to 40 years. Mean age at presentation was 36.45 years (range 12-70 years). A total of 14 male patients and 6 female patients formed this study group. Similar findings were reported from study conducted before,^29^ regarding incidence of age distribution and in their study, they reported the majority of cases were between 20 and 40 years. Among our patients, the most common etiology for soft tissue defect was trauma (9 cases, 45%) followed by 5 cases (25%), where flaps were used for post burn contracture reconstruction. In another study,^30^ regarding management of lower limb defects with propeller flap cover, they reported that most of soft tissue defects were caused by high energy acute trauma in the form of road traffic accidents, fall from height, industrial and direct trauma and similar findings were seen in our study. 

All patients, in addition to routine investigation, were submitted to Doppler examination for location of the perforators near the soft tissue defect. As stated, the position and caliber of cutaneous perforators are highly variable between individuals and often asymmetric even in the same individual. Therefore, Doppler evaluation was done twice, once pre-operatively and later to confirm location of perforator, intraoperatively. This combined with usage of an exploratory incision of perforator before raising flap was found prevents intra-operative setbacks of not finding appropriate perforators to design the flap upon intraoperatively. 

Ademola *et al.*^31^ used a similar approach, whereby they ensured presence, safety, and adequacy of the perforators used via pre-operative Doppler as well as the exploratory incisions before making incisions on the opposite sides of the flap. With regards to flap design, the commonest type of flap design used was longitudinal propeller flap design in 18 patients (90%). The range of flap movement varied with individual cases between 900-1800. The dimensions of perforator based propeller flaps ranged between 6x5 cm to 19×7 cm. The largest dimension of flap raised in this study was 19×7 cm (over the posterior tibial artery perforator for coverage of exposed ankle joint) and healed well without any complications. 

Regarding the largest possible dimensions of propeller flaps in various regions, many studies have been conducted. In one such study conducted by Panse *et al.*,^32^ an attempt to find a relationship between the necrosis rate of the flap and the rapport between the length of 74, the lower leg and the length of the flap were made and they concluded that there were six times more chance of necrosis for a length of the flap more than one-third of the limbs’ length. Koshima *et al.*^33^ reported used a posterior tibial artery propeller perforator flap of 19×13 cm successfully, where they extended the area of flap proximally, while still basing them on a single perforator and the largest flap in their experience was a posterior tibial artery propeller perforator flap of 28×13 cm which was consistent with our experience in this study. 

Our study assessed the clinical outcome of the flap in three categories and the findings were as follows: Majority of the cases (85%) had complete flap success (17 out of 20), while in 3 patients, the outcome was flap success with complications (15%). There were no patients who developed flap failure. Similar to our study results, Lazzeri *et al.*^34^ with their article published in 2013 found the overall flap survival rate to be 80.7%. With regards to successful utilization of propeller flaps for reconstruction in extremities, Masia *et al.*^35^ found a survival rate of 90% in their study, which is comparable to our results. They also concluded their study with the note that perforator flaps are safe and reliable flaps and represent an important step forward in reconstructive plastic surgery of the limbs. 

Whenever possible, surgical intervention and donor-site morbidity ought to be limited to a single body region, and the use of propeller perforator flaps can concretely widen the reconstructive options in extremities because of the advantages of (1) important decrease in donor-site morbidity, preserving muscles and their functions and sparing the main vascular trunks; (2) specificity in “1ike-to-like” soft tissue replacement; and (3) a better cosmetic and reconstructive result. 

The success rate in the present studies was very satisfactory with 95% flap take. There was 5% cases with total flap loss. Our study establishes the usefulness of this flap as an alternative to the free flap. After recovery, the patients could resume normal life. It could be concluded that careful selection of the perforator and optimal designing of the flap result in favorable outcomes with the use of local perforator flaps for reconstruction in extremities’. It provides predictable clinical outcomes with minimal donor site morbidity, is cost-effective, relatively easy technique and requires no special microsurgical setup or instruments. 

Hence, the present studies recommend the use of propeller perforator flaps as they allow reconstruction of even complex wound with local tissues in a single stage, accelerating recovery with minimal post-operative discomfort and frequently, a better aesthetic result than traditional methods of reconstruction. It was also observed that this surgery is easy and quick which is designed after marking the median sural artery and perforators with Doppler and the average operation time of elevating the flap was 30 minutes. 

No major arteries were sacrificed as it is a perforator based flap. Having a long arc of rotation, we could cover defects distally on the foot and heel. The blood supply was constant and reliable and the donor site deformity or morbidity was minimal. Though upper part of the sural nerve is cut, a part of the communicating posterior cutaneous nerve of the thigh remains, therefore, the sensation is preserved. It was observed that the younger patients in the age group of 10-30 regained sensation on the flap earlier than those of the older ones. As this is a fasciocutaneous flap, the durability is excellent in the weight bearing areas of the heel. After recovery, the patients treated with modified sural artery flaps and propeller preparator flaps could resume normal life.

## CONFLICT OF INTEREST

The authors declare no conflict of interest.
